# Phylotranscriptomic analysis uncovers a wealth of tissue inhibitor of metalloproteinases variants in echinoderms

**DOI:** 10.1098/rsos.150377

**Published:** 2015-12-04

**Authors:** Ronald M. Clouse, Gregorio V. Linchangco, Alexander M. Kerr, Robert W. Reid, Daniel A. Janies

**Affiliations:** 1 Department of Bioinformatics and Genomics, University of North Carolina at Charlotte, 9201 University City Boulevard, Charlotte, NC 28223, USA; 2 Marine Laboratory, University of Guam, Mangilao, GU 96913, USA; 3 ARC Centre of Excellence for Coral Reef Studies, James Cook University, Townsville, Queensland 4811, Australia; 4 Bioinformatics Services Division, North Carolina Research Campus, University of North Carolina at Charlotte, Kannapolis, NC 28081, USA

**Keywords:** collagen, Echinodermata, ECM, Holothuroidea, MCT, MMP

## Abstract

Tissue inhibitors of metalloproteinases (TIMPs) help regulate the extracellular matrix (ECM) in animals, mostly by inhibiting matrix metalloproteinases (MMPs). They are important activators of mutable collagenous tissue (MCT), which have been extensively studied in echinoderms, and the four TIMP copies in humans have been studied for their role in cancer. To understand the evolution of TIMPs, we combined 405 TIMPs from an echinoderm transcriptome dataset built from 41 specimens representing all five classes of echinoderms with variants from protostomes and chordates. We used multiple sequence alignment with various stringencies of alignment quality to cull highly divergent sequences and then conducted phylogenetic analyses using both nucleotide and amino acid sequences. Phylogenetic hypotheses consistently recovered TIMPs as diversifying in the ancestral deuterostome and these early lineages continuing to diversify in echinoderms. The four vertebrate TIMPs diversified from a single copy in the ancestral chordate, all other copies being lost. Consistent with greater MCT needs owing to body wall liquefaction, evisceration, autotomy and reproduction by fission, holothuroids had significantly more TIMPs and higher read depths per contig. Ten cysteine residues, an HPQ binding site and several other residues were conserved in at least 70% of all TIMPs. The conservation of binding sites and the placement of echinoderm TIMPs involved in MCT modification suggest that ECM regulation remains the primary function of TIMP genes, although within this role there are a large number of specialized copies.

## 1. Introduction

Tissue inhibitors of metalloproteinases (TIMPs) in animals are important regulators of the extracellular matrix (ECM), which is a complex and dynamic arrangement of various molecules [1]. Early studies in humans characterized TIMPs primarily as inhibitors of metalloproteinases (MMPs or ‘matrixins’) [2,3], but they are increasingly understood to have a variety of regulatory and biosynthetic functions [1] and in some cases may interact directly with ECM proteins [4]. The role of TIMPs in modifying the ECM may give them important applications in materials science [5] and the treatment of cancer [1,6].

**Figure 1.**
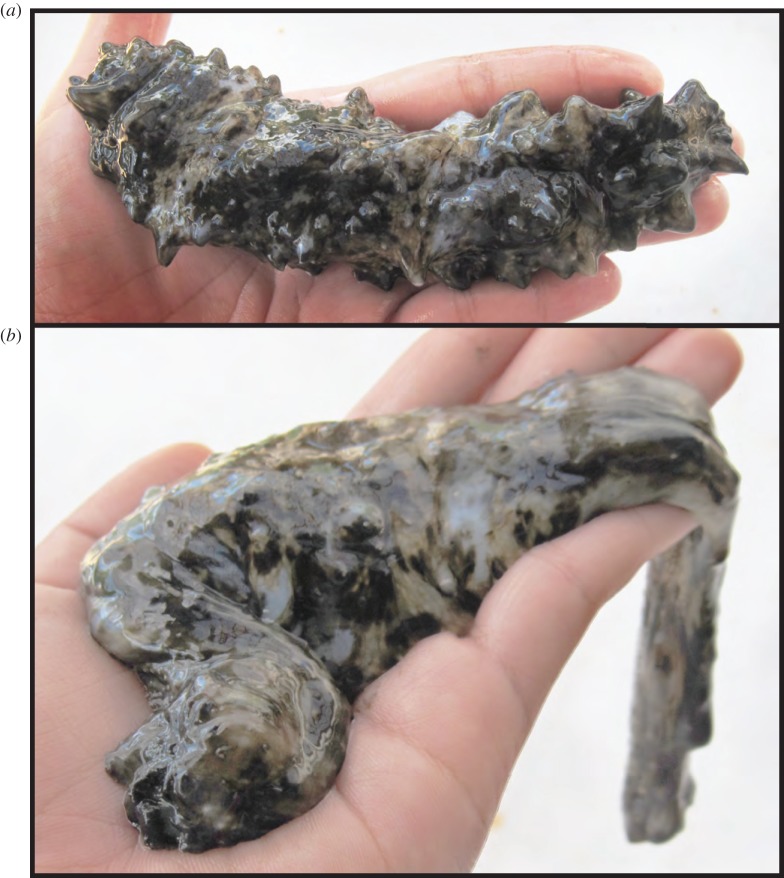
*Stichopus horrens* Selenka, 1867 before (*a*) and after (*b*) dermal liquefaction (photos: Katherine Brunson).

Echinoderms are known for rapidly altering the tensile strength of certain collagenous tissues after various stimuli [5,7,8], a process that that has been associated with the presence of certain TIMPs. These tissues are thus referred to as ‘mutable collagenous tissues’ (MCTs) [5], ‘catch connective tissues’ [7] or ‘mutable connective tissues’ [9], and they are apparently under nervous control [5]. MCT is also found in the mammalian cervix, and its modelling during pregnancy and birth has been shown to be influenced by various MMPs and TIMPs [10]. MCT liquefaction in the body walls of holothuroids (sea cucumbers) is a dramatic (but reversible; figure 1) response to danger that permits autotomy and ballooning when escaping predators [11]. Two TIMP-like molecules identified from holothuroids—tensilin (or *C*-tensilin) [12] and *H*-tensilin [13]—have shown the specificity of TIMP functions in MCT modification, for both of these cause collagenous tissue to transform only from a soft to a standard level of stiffness. Different factors stiffen the tissue even further and reverse the effects. Less visible than holothuroid body-wall liquefaction, but critically important, is the role of MCT in the functioning of the echinoid (sea urchin) feeding apparatus [14,15]. These MCT ligaments demonstrate three levels of stiffness (reminiscent of holothuroid MCT) [15] and respond to changes in concentrations of MMPs, an echinoid tensilin-like protein and a synthetic MMP inhibitor (although only weakly in the latter two cases) [14,15].

TIMPs have also been identified as important inhibitors of metastasis and tumour growth, mostly because of their role as inhibitors of MMPs, which renew and modify the ECM [1]. The process of ECM modification is important for generating conditions favourable to colonizing cancer cells and the development of their vasculature. Several MMP family members are also directly involved in signalling pathways that regulate angiogenesis, inflammation and cell proliferation. Thus, scenarios which increase the ratio of TIMPs to MMPs are associated with decreased metastasis and tumour growth [1,2,13,16–18]. Moreover, TIMP-2 appears to suppress cancer growth with mechanisms independent of MMP regulation [19]. An open question is whether non-human TIMPs can give insights into the mechanisms of cancer inhibition or, more directly, provide effective therapeutics. As fellow deuterostomes, echinoderms should be the first point of comparison with vertebrates as a potentially fruitful ground for bioprospecting for TIMPs.

We saw an opportunity to significantly advance our understanding of TIMPs by generating a phylogeny from echinoderm TIMP transcripts; this would test the importance of TIMPs in the development of MCT in echinoderm evolution and identify which echinoderm TIMPs are most closely related to those from vertebrates. Because, unlike other echinoderms, most holothuroids rely on MCTs to maintain body turgor [7], and they perform complex soft-tissue modifications such as evisceration and whole-body fission [7], we hypothesized that we would find large clades of holothuroid TIMPs with large read depths, and that they would have evolved after that class’s split from their sister, the echinoids (sea urchins) [20]. Moreover, a holothuroid basal sister group, the Apodida [21], which has thin body walls and may rely on coelomic pressure more than MCTs for body turgour [22], was hypothesized to have the fewest number of TIMP genes and the smallest read depths. Some invertebrate TIMPs have been identified as vertebrate TIMP-1 or TIMP-2 homologues already, so we tested whether echinoderm TIMPs would be recovered among clades of vertebrate TIMPs 1–4. To date, key questions about TIMPs have concerned their functional characterization, such as the identification of binding sites and their ligands [2], so we also examined the conserved domains to assist further studies on their biochemical and material properties.

## 2. Material and methods

### 2.1 Tissue preparation, sequencing and assembly

Forty-one echinoderm specimen samples (tube feet, pinnules or body-wall tissue from adults) were preserved in Qiagen RNAlater^r^ (Qiagen, Valencia, CA, USA), and RNA extractions were quantified using an Agilent 2100 BioAnalyzer (Agilent Technologies, Santa Clara, CA, USA). RNA was purified using the standard protocols of Qiagen miRNeasy kits. The specimens represented at least 40 species, 24 orders and 37 families in all five extant classes (table 1). One *Psolus* specimen was a juvenile which we were unable to determine as a different species from the other *Psolus*, which is undescribed, and the two are treated as different species here. One specimen was used for each sample listed in table 1, and species were selected so as to represent specific clades and span as much of the evolutionary diversity of the phylum as possible. Tissues were sampled so as to maximize fully differentiated, adult, soft tissue. Specimen vouchers are deposited in Scripps Institution of Oceanography, UC San Diego, Benthic Invertebrates collection. Animal treatment met recommended ethical standards for invertebrates; collection permits are on file with the authors and available upon request. Extractions were subjected to RNA-Seq sequencing on an Illumina Hiseq 2000 platform (100 BP, paired end). Reads for each of the samples were filtered by quality score (cut-off threshold>Q20) by fastxtrimmer, and Illumina adapters were then removed by fastxclipper; these are both components of the FASTX toolkit v. 0.0.13.2 [23].

**Table 1.** The number of contigs and reads, both in total and for the 405 TIMP genes identified here, for all taxa sampled. TIMP contigs and reads are also shown as a proportion of all contigs and reads, and the ranking of these proportions are shown.

	contigs	reads	TIMPs	TIMP reads	avg. reads/TIMP contig	% TIMPs	rank	% TIMP reads	rank
Asteroidea (*n*=14)									
*Asteropsis carinifera*	49 607	30 392 805	6	5491	915.2	0.012	33	0.018	21
*Astropecten duplicatus*	73 745	29 277 239	11	2023	183.9	0.015	29	0.007	27
*Cheiraster hirsutus*	1271	2 159 361	0	0	n.a.	0.000	39.5	0.000	39.5
*Henricia* cf. *leviuscula*	76 684	29 546 206	10	7390	739.0	0.013	30	0.025	16
*Labidiaster annulatus*	40 071	112 755 029	9	12 054	1339.3	0.022	24	0.011	26
*Luidia clathrata*	77 487	28 501 794	5	4918	983.6	0.006	37	0.017	22
*Odinella nutrix*	1004	4 134 835	0	0	n.a.	0.000	39.5	0.000	39.5
*Peribolaster folliculatus*	57 013	69 119 595	15	2459	163.9	0.026	19	0.004	32
*Pisaster ochraceus*	43 479	139 502 413	13	19 690	1514.6	0.030	15	0.014	23
*Glabraster antarctica*	54 328	21 039 565	7	706	100.9	0.013	31	0.003	33
*Psilaster charcoti*	28 413	46 739 511	9	10 505	1167.2	0.032	13	0.022	17
*Pteraster tesselatus*	51 764	142 489 961	13	138 738	10 672.2	0.025	21	0.097	8
*Remaster gourdoni*	22 056	36 388 063	5	1819	363.8	0.023	23	0.005	30
*Xyloplax janetae*	24 452	20 903 794	3	359	119.7	0.012	32	0.002	34
mean:	42 955	50 925 012	7.6	14 725	1521.9	0.016	27.6	0.016	26.3
Crinoidea (*n*=9)									
*Cenolia* n. sp.	18 875	13 882 168	5	7085	1417.0	0.026	18	0.051	13
*Democrinus brevis*	8287	23 656 961	6	26 569	4428.2	0.072	8	0.112	7
*Gephyrocrinus messingi*	12 234	24 58 898	2	2811	1405.5	0.016	27	0.012	25
*Isometra vivipara*	43 689	28 460 271	9	13 707	1523.0	0.021	26	0.048	14
*Oligometra serripinna*	70 278	156 085 944	20	90 031	4501.6	0.028	16	0.058	11
*Phrixometra nutrix*	11 855	13 923 251	3	6404	2134.7	0.025	20	0.046	15
*Promachocrinus kerguelensis*	12 283	10 362 958	6	14 833	2472.2	0.049	12	0.143	6
*Psathryometra fragilis*	9015	27 444 619	0	0	n.a.	0.000	39.5	0.000	39.5
*Ptilometra australis*	49 470	27 016 836	14	42 525	3037.5	0.028	17	0.157	4
mean:	26 221	36 121 323	7.2	22 663	2614.9	0.030	20.4	0.070	14.9
Echinoidea (*n*=5)									
*Arbacia punctulata*	33 220	16 672 722	3	1142	380.7	0.009	36	0.007	28
*Dendraster excentricus*	12 561	13 766 000	3	167	55.7	0.024	22	0.001	35
*Echinaster spinulosus*	18 608	31 429 052	2	31	15.5	0.011	35	0.000	37
*Eucidaris tribuloides*	16 512	11 565 762	5	610	122.0	0.030	14	0.005	29
*Strongylocentrotus purpuratus*	11 368	11 237 474	7	2075	296.4	0.062	10	0.018	20
mean:	18 454	16 934 202	4.0	805	174.1	0.027	23.4	0.006	29.8
Holothuroidea (*n*=9)									
*Abyssocucumis* sp.	26 171	13 434 829	32	21 824	682.0	0.122	3	0.162	3
*Molpadia intermedia*	6516	18 146 083	7	2222	317.4	0.107	5	0.012	24
*Peniagone* sp.	22 457	21 508 002	24	32 436	1351.5	0.107	6	0.151	5
*Pannychia moseleyi*	20 051	19 231 829	13	16 855	1296.5	0.065	9	0.088	9
*Pseudostichopus*sp.	5567	10 992 832	3	540	180.0	0.054	11	0.005	31
*Psolus*sp.11	35 310	42 192 822	45	34 748	772.2	0.127	2	0.082	10
*Psolus*sp.41	33 062	27 131 787	45	14 600	324.4	0.136	1	0.054	12
*Stichopus chloronotus*	24 854	54 588 991	20	297 835	14 891.8	0.080	7	0.546	2
*Synapta maculata*	11 154	47 675 387	13	535 956	41 227.4	0.117	4	1.124	1
mean:	20 571	28 322 507	22.4	106 335	6782.6	0.102	5.3	0.247	10.8
Ophiuroidea (*n*=4)									
*Astrophyton muricatum*	26 889	17 829 133	3	3630	1210.0	0.011	34	0.020	19
*Ophiocoma wendtii*	9783	5 810 700	0	0	n.a.	0.000	39.5	0.000	39.5
*Ophioderma brevispinum*	28 450	17 347 877	6	3535	589.2	0.021	25	0.020	18
*Ophiothrix spiculata*	18 816	20 275 162	3	79	26.3	0.016	28	0.000	36
mean:	20 985	15 315 718	3.0	1811	608.5	0.012	31.6	0.010	28.1

RNA-seq produced a total of 2 360 841 332 raw reads and ranged per sample from 30 190 658 (*Cheiraster* sp.) to 88 987 394 (*Pisaster ochraceus*). Following trimming and adapter removal, 2 101 192 636 reads remained. This was a reduction of approximately 11% in total, and ranged per sample from 3.65% (*Gephyrocrinus messingi*) to 19% (*Promachocrinus kerguelensis*). A correlation between taxonomic class and read count was not observed. De novo assembly of contigs was then performed using Trinity [24,25] on a high-memory computer cluster using 500 GB of RAM and 24 CPUs. Contigs for each sample were translated using Transdecoder (http://transdecoder.sourceforge.net) and the PFAM-B protein family database [26] (minimum protein length=100).

### 2.2 Protein characterization and tissue inhibitor of metalloproteinases identification

An Access^^TM^^ database was constructed to contain the transcriptomes for all species. For each entry, there was an amino acid sequence (‘*aa*’), the underlying coding sequence (‘*cds*’) and the original raw nucleotide sequence (‘*nuc*’). Each putative protein was annotated using InterProScan [27,28], which searches for protein signature matches in the InterPro database [29], as well as searches against the PANTHER [30,31], Pfam [26] and PROSITE [32] databases. Those 405 transcripts identified as TIMPs in these databases were then exported; they have also been deposited in GenBank under accession nos. KT935675–KT936079 and are available in the electronic supplementary material and at the Dryad Digital Repository (datadryad.org,). Five publicly available echinoderm TIMP sequences were then added, four from the annotated genome of the echinoid *Strongylocentrotus purpuratus* (GenBank accession no. XM_003725476, XM_001198302, XM_775549 and XM_003725477) and tensilin from the holothuroid *Cucumaria frondosa* (GenBank accession number AY033934). GenBank was also searched for non-echinoderm TIMP genes, and for each of those 71 unique entries the full nucleotide, coding and amino acid sequences were downloaded and added to those from the echinoderm transcriptome database. At this stage, each of the *aa*, *cds* and *nuc*alignments had the same 481 terminals.

### 2.3 Alignment, culling and tree-searching

We then began a process of recursively aligning, examining and culling our collection of TIMPs (independently for *aa*, *cds* and *nuc* sequences; electronic supplementary material, figure S1). Despite having some subregions identified as TIMP genes when analysed by protein databases, we questioned whether highly divergent contigs were homologous to our core dataset. With no other tests of homology available to us, highly dissimilar contigs were considered possible artefacts and thus removed. Highly divergent sequences, especially those with large indels, also had the potential to be disruptive to alignment algorithms, even if truly homologous.

The data were aligned in MAFFT [33] (using the *localpair* option and default settings for other parameters), and a distance matrix was generated in BioEdit [34]. This dataset was examined for highly divergent contigs, which were discovered to be partial sequences among the GenBank downloads, as well as the human TIMP-2 sequence, all of which were removed. We later discovered that human TIMP-2 aligned reasonably after further culling (described below), and it was included in the final phylogeny. Next, we identified identical sequences among the *nuc* alignments (again, all among the GenBank downloads) and removed them from the *aa*, *cds* and *nuc* alignments.

To increase the quality of the alignments prior to tree-searching, we used a two-step process that employed the program trimAl [35] and a custom python script we call ‘Boxer’. trimAl enables the removal of difficult-to-align sequences via an automated command–line interface employing alignment statistics. Boxer selects from alignments produced by trimAl, preferring those with the largest number of unique taxa given a maximum percentage of gaps in the entire alignment. trimAl identifies difficult-to-align sequences using two measurements of a preliminary alignment: (i) ‘residue overlap’, which is the proportion of an alignment column occupied by residues (not gaps or missing data) and (ii) ‘sequence overlap’, which is the percentage of positions with residues (not gaps or missing data) in an aligned sequence. If a sequence does not fulfil both of the user-set thresholds for these parameters, it is removed from the alignment and the data realigned and evaluated. This process was performed using six settings (50%, 60%, 70%, 80%, 90% and 100%) for each overlap parameter, giving us 36 alignments from which to continue. We chose the alignment for each of the three sequence types that had the largest reduction in gaps while also retaining at least one of the sequences downloaded from GenBank for *Branchiostoma floridae*, a cephalachordate and a possible source of important transitional sequences between echinoderm and the vertebrate TIMP sequences. The chosen alignments from Boxer had the following total number of terminals: *aa* 373 (294 echinoderm), *cds* 406 (327 echinoderm) and *nuc* 319 (246 echinoderm).

For each of the three sequence types, we then did another round of terminal reduction based on stability in trees. We conducted rapid bootstrapping tree-searches in RAxML [36], rooting by a sequence from the bivalve *Crassostrea gigas* (GenBank accession number AF321279). We then uploaded bootstrap trees and the best tree to RogueNaRok [37], an online application for identifying ‘rogue’ or ‘bouncing’ terminals. Terminals were then removed from the alignment if this caused the sum of support values on the best tree to improve by 0.2 or more. From the amino acid alignment, 10 terminals were removed, 11 from *cds* and 12 from *nuc*. The gastropod *Haliotis diversicolor* (GenBank EU244343) was removed from all three alignments, but most of the other removed rogue terminals were unique to the different alignments.

The newly reduced sequence sets were aligned again in MAFFT, this time using the *localpair* strategy for the *aa* and *cds* alignments and *globalpair* for the *nuc* alignment (which is better at handling large indels). They were then subjected to rapid bootstrap maximum-likelihood searches in RAxML. The alignments output by Boxer, chosen using the *B. floridae* rule and then reduced further in RogueNaRok, included four protostomes (all bivalves), which were all in the *aa*, *cds* and *nuc* alignments. Final trees were read into R [38] and compared using the packages *ape* and *dendextend* [39]; the ‘tanglegram’ command in the latter package was used to visualize the stability of results from the three different sequence types.

Finally, we chose a representative sequence for each taxon in each of the many small clades of similar sequences, avoiding those with small read depths, and we added back the human TIMP-2 sequence and *Drosophila melanogaster* sequences (culled earlier). We considered the latter two important sequences to be analysed in this study (and they aligned reasonably in the smaller dataset), but we could not find a reasonable alignment for the TIMP gene from *Caenorhabditis elegans* and did not include it. Sequences were more likely to be eliminated (especially from the *nuc* dataset) during culling if they had large read depths, so we also returned a holothuroid sequence culled previously from *aa*, *cds* and *nuc* datasets with the second highest read depth (a *Synapta maculata* contig based on 240 066 reads) to examine its behaviour in tree searches. This sequence was actually conserved at key TIMP positions, but it also had a long C-terminal chain (over 100 amino acids) not shared by other sequences. We also removed an asteroid (sea star) sequence that had been recovered among vertebrate TIMP-2 sequences; it was identical to mouse TIMP-2 for part of the sequence, and given its low read depth (18) we considered it a contaminant. This final alignment of coding sequences, consisting of 180 echinoderm and 46 non-echinoderm sequences, was then subjected to rapid bootstrapping tree searches in RAxML.

### 2.4 Diversification rates and conserved domains

To examine diversification rates of TIMPs in different species, especially the apodan holothuroid *S. maculata*, we analysed lineages-through-time (LTT) plots using the R package *ape*. To perform this analysis, we first took the alignment of coding sequences from Boxer which had the largest reduction in terminals while still retaining 10 or more *S. maculata* sequences. This was the alignment of 463 copies, 392 of which were echinoderm. We then found the most likely tree by using RAxML with the same settings described above, and we made the resulting tree ultrametric with the program PATHd8 [40]. PATHd8 is used to date phylogenies by adjusting node heights based on user-input date parameters, and it is considered particularly effective for large phylogenies. We fixed the root to ‘100’ (arbitrarily, because this number does not factor into relative node height adjustments absent from other inputs). The ultrametric tree was then read into R, and all terminals but those of interest (those specimens with 10 or more TIMPs) and the two *C. gigas* outgroups were pruned using the ‘drop.tip’ command. Each of the new trees (one for each taxon of interest) was then used to make LTT plots, and a gamma statistic [41] was calculated for each. *p*-Values for gamma statistics were calculated using the command ‘2*(1-pnorm(abs(gammaStat(phylo))))’, where ‘phylo’ is the name of the tree read into the memory. LTT plots with larger gamma statistics have diversification patterns more divergent from a constant rate of growth.

To examine the conserved domains across animal TIMPs, we took the final alignment of 226 TIMP amino acids and made consensus sequences showing those residues found in at least 90%, 80%, 70%, 60% and 50% of all sequences, counting gaps as sites. For the protostomes, chordates and each of the five classes of echinoderms, we made consensus sequences at the 70% threshold for comparison.

## 3. Results

**Figure 2.**
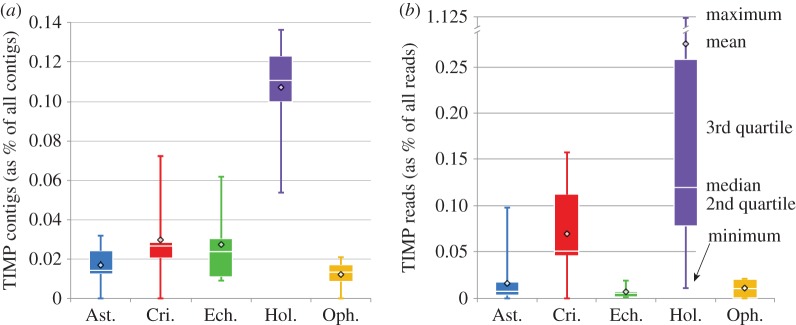
Boxplots for the 405 TIMP genes identified in our transcriptome database, represented as a proportion of all contigs assembled from each taxon, and averaged across each of the five classes (*a*), as well as the average read depth per TIMP contig, as a proportion of all reads for each taxon, averaged for each class (*b*).

The number of TIMP genes per sampled taxon, as a proportion of all contigs from that taxon, was significantly different among the five classes (Kruskal–Wallis, d.f.=4, *p*=0.0001), using our randomly selected, single exemplars of the chosen species from each class. The average proportion in holothuroids was about three to eight times higher than in the other classes (figure 2 and table 1). The number of reads per TIMP contig, as a proportion of all reads from that taxon, also differed among the sampled taxa (Kruskal–Wallis, d.f.=4, *p*=0.003), with the average proportion in holothuroids being 4–39 times this proportion in the other classes. Ophiuroids had the lowest proportion of TIMP genes per taxon (0.012%), and echinoids had the lowest proportion of reads per TIMP gene (0.006%).

The average read depth of full nucleotide sequences which were culled (7446.4±95% CI: 4628.4) was greater than those kept through the process (652.3±279.8; two-tailed *t*-test, unequal variances, *p*=0.005). The effect was more pronounced in holothuroid sequences (culled=11 469.4±9712.9 versus kept=591.00±225.16). There was no difference in read depths between culled and kept amino acid and coding sequences.

The proportions of TIMP genes and read depth varied among holothuroid taxa, but our apodan (*S. maculata*) did not have fewer TIMPs than other holothuroids. The proportion of TIMP contigs in *S. maculata* was 0.117%, second only to the dendrochirotids (*Abyssocucumis* and *Psolus*, 0.129%), and the proportion of reads in TIMP contigs was higher in *S. maculata* (1.124%) than all other taxa, including those in other classes. Indeed, two *S. maculata* TIMP contigs had depths of over 200 000 reads.

Optimal trees from the *aa*, *cds* and *nuc* terminal sets produced by our alignment and culling procedure (−lnl=75 586.54, 148 089.55 and 187 240.12, respectively) consistently recovered the vertebrate TIMPs as monophyletic, and within that clade each of the four TIMP types (1–4) also as monophyletic (electronic supplementary material, figures S2 and S3). Sequences characterized as TIMP-1 or -2 outside of vertebrates (i.e. in cephalochordates and protostomes) appear to have no relationship with vertebrate TIMP-1 or -2 genes. The cephalochordate sequences included (i.e. from *B. floridae*) were recovered as sister to the vertebrate ones with the *aa* and *nuc* terminal sets, and separated from the vertebrate clade by three unstable echinoderm ones in the *cds* terminal set. Crinoid sequences consisted mostly of the older TIMP lineages, and holothuroid sequences dominated more recent diversifications. All trees had low bootstrap support for relationships among the different echinoderm and chordate lineages, but increasing support among the more derived clades.

**Figure 3.**
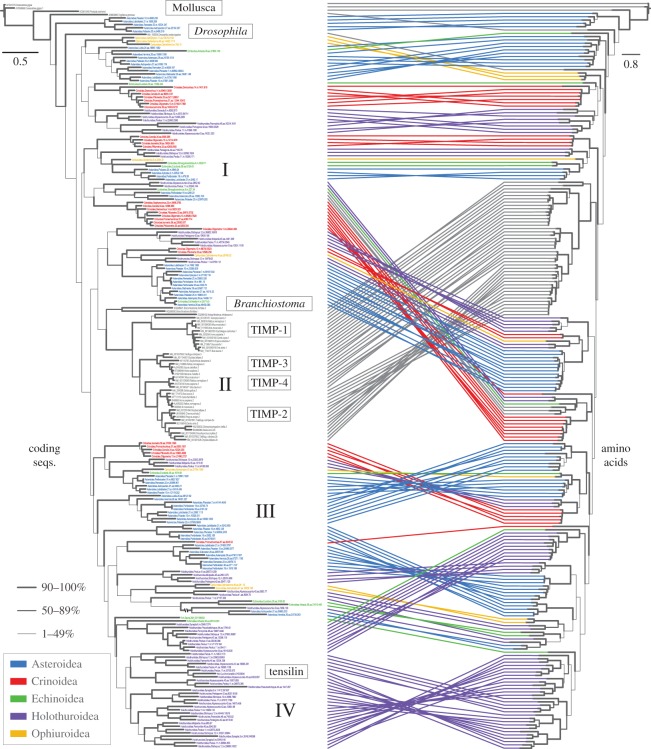
Best tree recovered under maximum-likelihood using the final, selected terminal set of 180 echinoderm and 46 non-echinoderm sequences. They were aligned as amino acids then back-translated; the tree to the left was recovered from using coding sequences, and the tree to the right shows the result of tree-searching the same alignments translated to amino acids. Numbers I–IV denote key events in the evolution of deuterostome TIMPs, shown in figure 5.

Our tree from the final terminal set of coding sequences (−lnl=128 878.97)—culled of redundant clade representatives and with human TIMP-2 and other important sequences re-added—showed the same patterns as the more inclusive trees: monophyletic chordate, vertebrate and TIMP-1–4 sequences, an early diversification of TIMPs, a diversification after vertebrates (mostly in holothuroids), and low bootstrap support among constituent lineages (figure 3). The *D. melanogaster* sequence was recovered among the earliest diverging echinoderm lineages (which were mostly asteroid), and the human TIMP-2 sequence was recovered among other vertebrate TIMP-2 sequences. The re-added holothuroid sequence with the second highest read depth was recovered within a recent diversification of holothuroid sequences and sister to a sequence from the same taxon. This clade of holothuroid sequences was recovered as sister to echinoid sequences. The translation of the final coding sequence alignment recovered a very similar tree (−lnl=75 400.75), although it placed the chordate TIMPs (which had bootstrap support >50%) earlier in the tree than the coding sequences (figure 2).

**Figure 4.**
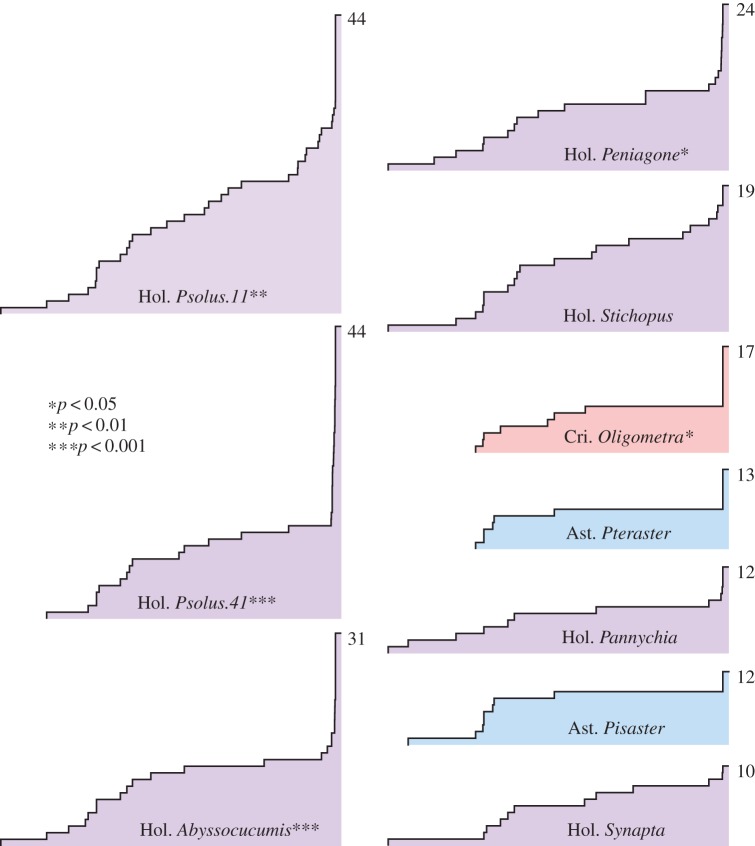
Simplified lineages-through-time (LTT) plots for TIMP genes in specimens with more than 10 copies in an alignment of 392 echinoderm TIMP coding sequences. The *x*-axis of each plot is the time since the root, and the *y*-axis is the number of lineages; the final lineage count is shown to the right of the final height. *p*-Values for the gamma statistic are shown for each specimen, lower values indicating a greater deviation from a constant rate of diversification.

Lineages-through-time plots showed a significant recent acceleration in the accumulation of TIMP copies in four holothuroids (*Psolus* sp. 11, *Psolus* sp. 41, *Abyssocucumis* sp. and *Peniagone* sp.) and one crinoid (*Oligometra serripinna;* figure 4). *Stichopus chloronotus* had the fifth largest number of TIMPs in the coding sequence alignment we examined (19) but had acquired them through a steady accumulation of copies over time. The remaining taxa with 10 or more TIMPs in this alignment (the asteroids *Pteraster tesselatus* and *P. ochraceus* and the holothuroids *Pannychia moseleyi* and *S. maculata*) had the smallest number of TIMPs (between 10 and 13) and showed no significant change in the rate of their accumulation over time.

Of the 12 cysteine residues previously reported as conserved in vertebrate TIMPs [42], 10 are found in 70% of all TIMPs (table 2). TIMPs appear to retain 12 cysteine residues in general, but the locations of those at the C-terminus vary in location among different taxa. The ‘VIRAK’ motif identified in vertebrates [42] is not conserved outside of chordates, but looking at our original download of 405 echinoderm TIMPs, we found it in one asteroid copy (from *Labidiaster annulatus*), 10 crinoid copies (four from *Oligometra serripinna* and six from *Ptilometra australis*) and two holothuroid copies (both from *S. maculata*). An SA-binding motif (HPQ) is highly conserved across all TIMPs (including chordates), found in at least 70% of all copies in our final alignment. Other residues conserved in 70% of all TIMPs, located across positions 320–596 of our final, 833-position alignment, are four glycines (G), three tyrosines (Y) and one each of lysine (K), threonine (T), leucine (L), glutamine (Q) and tryptophan (W) (table 2).

**Table 2.** TIMP consensus sequences, with various terminal groups and consensus thresholds, using the final, selected terminal set of 226 echinoderm and outgroup TIMPs. Location in the 833-position alignment is shown in the top row. Conserved positions shown are all cysteine residues, the VIRAK motif identified earlier in vertebrates (positions 174–178), and any conserved residues on the 70% consensus of all TIMPs.

terminals	threshold (%)	150	153	162	164	165	169	174	175	176	177	178	320	334	335	362	372	375
chordates	70	C	C	H	P	Q	C	V	I	R	A	K	Y	K	G	T	C	G
protostomes	70	C	C	H	P	Q	C	V	—	—	—	—	Y	K	—	T	C	G
Asteroidea	70	—	C	H	—	Q	C	V	—	—	—	—	Y	K	G	—	C	G
Crinoidea	70	—	C	H	P	Q	C	—	I	R	—	—	Y	K	G	T	C	G
Echinoidea	70	—	—	—	—	—	—	—	—	—	—	—	Y	K	—	—	C	G
Holothuroidea	70	C	C	H	P	Q	C	—	—	—	—	—	—	K	—	T	C	G
Ophiuroidea	70	—	—	—	—	—	—	—	—	—	—	—	—	—	—	—	C	G
all	90	—	—	—	—	—	—	—	—	—	—	—	—	—	—	—	C	—
all	80	—	C	—	—	—	C	—	—	—	—	—	—	K	—	—	C	G
all	70	C	C	H	P	Q	C	—	—	—	—	—	Y	K	G	T	C	G
all	60	C	C	H	P	Q	C	V	—	—	—	—	Y	K	G	T	C	G
all	50	C	C	H	P	Q	C	V	I	—	—	—	Y	K	G	T	C	G

terminals	threshold (%)	380	396	400	440	456	459	475	479	484	493	498	548	572	579	594	596	
chordates	70	—	Y	G	C	Q	—	Y	C	C	C	C	C	—	C	C	W	
protostomes	70	—	Y	G	C	Q	G	Y	C	C	C	C	C	C	C	C	W	
Asteroidea	70	L	Y	G	C	Q	G	Y	C	C	—	—	C	C	C	C	W	
Crinoidea	70	L	Y	G	C	Q	G	Y	C	C	C	—	C	C	C	C	W	
Echinoidea	70	L	Y	G	C	—	—	—	—	—	—	—	—	—	—	—	—	
Holothuroidea	70	—	Y	G	C	Q	—	—	C	C	—	—	C	C	C	C	W	
Ophiuroidea	70	—	Y	—	C	—	—	Y	—	—	—	—	—	—	—	—	—	
all	90	—	—	—	C	—	—	—	C	—	—	—	—	—	—	—	—	
all	80	—	Y	G	C	Q	—	Y	C	C	—	—	C	—	—	C	W	
all	70	L	Y	G	C	Q	G	Y	C	C	—	—	C	—	C	C	W	
all	60	L	Y	G	C	Q	G	Y	C	C	—	—	C	C	C	C	W	
all	50	L	Y	G	C	Q	G	Y	C	C	C	—	C	C	C	C	W	

## 4. Discussion

TIMP genes are far more numerous and diverse in structure than was previously known [1], and our multiple phylogenetic analyses displayed a consistent pattern: an early diversification of TIMPs, many of which were retained in echinoderms; monophyletic chordate, vertebrate and TIMP-1–4 sequences; and continued TIMP diversification, especially in holothuroids, after the evolution of those in vertebrates.

**Figure 5.**
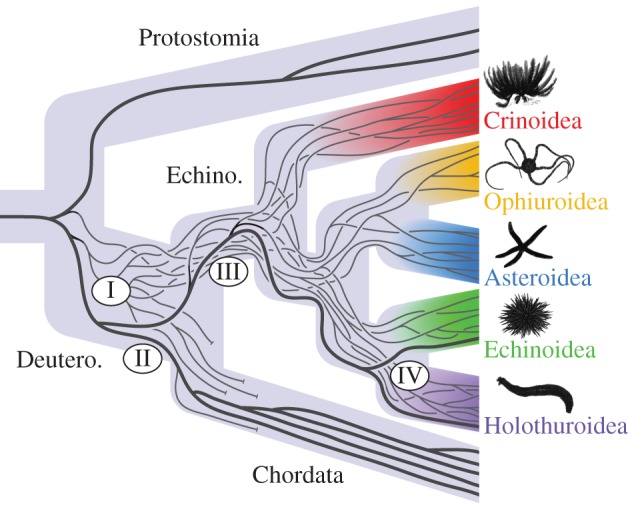
Schematic of key events in the evolution of TIMP genes in deuterostomes. After the split between Protostomia and Deuterostomia, there was a diversification of TIMPs which persisted into the five echinoderm classes (I). One early TIMP lineage was inherited by the ancestor of all chordates and is the progenitor of TIMPs 1–4 studied in vertebrates (II), and its sister lineage diversified and continued into the echinoderms (III). Of this latter lineage, one copy diversified much later in holothuroids, giving rise to the tensilin gene (IV).

Through continual diversification, echinoderms have greatly expanded their repertoire of TIMP genes, but, unlike chordates, they have also retained ancient copies (figures 3 and 5). Of the metazoan classes examined, holothuroids have the most TIMP copies, and holothuroid contigs have the highest read depths (figure 2 and table 1), supporting the idea that MCT has been extremely important in their evolution. The apodan holothuroid *S. maculata*, which was hypothesized to have the fewest TIMPs among holothuroids owing to its extremely thin body wall [22], has the fourth highest number of TIMP genes per contig (0.117%) and uses them heavily (table 1). This is a result that does not support the hypothesis that TIMPs increased in number in holothuroids as a response to their evolution of extensive MCTs. However, *S. maculata* is the only holothuroid we found to have any TIMPs with the VIRAK domain conserved in chordate copies, and *S. maculata* did not acquire its TIMP diversity through a recent radiation (figure 5). This suggests that apodans rely on an older suite of TIMPs that resemble and may perform many of the same functions as vertebrate TIMPs. It may be that apodans, all echinoderms and even chordates use some TIMPs to control the mechanical properties of specific ligaments and membranes within their bodies, as has been documented in echinoids [14,15].

The finding of recent radiations of holothuroid TIMPs as well as highly divergent holothuroid copies with high read depths does suggests that holothuroids have evolved specialized TIMPs, some of which they employ heavily. These divergent copies retain important TIMP characteristics, and the culling of many of them in our methods was most likely the result of large indels confounding alignment programs. The finding of tensilin in the same clade as the modern holothuroid TIMPs, including one of these highly divergent copies, implies that ECM modification has remained an important function throughout the history of TIMPs, including the new copies in holothuroids. Some of these may be used during evisceration, and perhaps they reflect modifications necessary for holothuroids to maintain MCT functioning as they evolved new morphologies and diversified into a wide array of environments. In addition, holothuroids, including apodans, autotomize, as do other echinoderms, but in holothuroids this is a complex process that nearly destroys corporal integrity. Moreover, some holothuroids reproduce via fission, another radical remodelling of numerous soft tissues. Controlling fission and autotomy may have selected for a deep and varied TIMP toolkit in holothuroids, the evolution of which is clearly visible in our phylogeny.

Despite having nearly 10 times the number of species, chordates have one-sixth the number of TIMPs sequenced as we now have from echinoderms. Chordate TIMPs appear to have descended from a single copy in their ancestor, which also lost the older copies currently being used in echinoderms. The current arrangement of vertebrate TIMPs into four types is supported by our phylogeny (figure 3), but the taxonomy of vertebrate TIMPs is inapplicable to invertebrates.

The large number of echinoderm TIMPs we uncovered here may explain why a tensilin-like gene identified from *S. pupuratus* [15] and galardin (a synthetic TIMP) [14] had only a weak effect on echinoid MCT. We found 20 echinoid TIMPs, including seven different sequences in *S. purpuratus*. This suggests a degree of specificity in TIMPs, and a productive avenue for future research would be to understand how our different transcripts map to functionally different proteins and how their expression changes across tissues and developmental stages.

One question that immediately arises is whether the vast array of echinoderm TIMPs has any relevance to studies of TIMPs as important ECM modulators in vertebrates, because these copies are separated by millions of years of evolution. However, TIMPs from across the tree show conservation in binding sites, with most copies having almost all the same cysteine residues, an HPQ binding site and various other highly conserved domains. The HPQ domain is shown in our final alignment with a position between the H and P (positions 162 and 164, respectively), but this is an artefact of the alignment; all copies that had this domain had the three elements in tandem. If vertebrate TIMPs do have unique functions in deuterostomes, it may perhaps be due to their VIRAK domain, but even this has been conserved in some echinoderm copies.

The echinoderm TIMPs identified here offer us a large, diverse pool of naturally occurring TIMPs that can inform theoretical and practical studies of these important genes. The goal now is to bridge the gap between fine-scale studies of specific tissues and molecules and our new understanding of how abundant and varied TIMPs really are. The former classification of TIMPs into four types is clearly applicable only to vertebrates, and what is desired now is a much broader functional classification that will assist in understanding the roles of various TIMPs in the many different processes they influence. For example, do certain domains indicate direct interaction with ECM proteins versus MMP inhibition? Are there consistent characteristics among those TIMPs involved in tumour suppression, and what are they? General principles of TIMP functioning would emerge from the building of a more comprehensive classification system, and this would be a key prerequisite to fabricating TIMP-like molecules useful in tumour suppression or the activation of MCT-like materials.

## Supplementary Material

Fig.S1.pdf: This is a figure that shows the workflow of the study.

## Supplementary Material

 Fig.S2.pdf: This is a comparison of trees that resulted from searching on alignments of amino acids, coding sequences, and raw nucleotide sequences.

## Supplementary Material

Fig.S3.pdf: This is the most likely tree found using coding sequences, the alignment of which resulted from our initial alignment-and-culling procedure.

## Supplementary Material

TIMP_sequences.nexus: This is a nexus file that contains the sequence data used in this study.

## Data accessibility

Transcripts identified as TIMPs have been deposited in GenBank under accession nos. KT935675–KT936079 and are available in the electronic supplementary material and at the Dryad Digital Repository (datadryad.org, http://dx.doi.org/10.5061/dryad.vt0j1).
