# Supplementary material for: Phylotranscriptomic analysis uncovers a wealth of tissue inhibitor of metalloproteinases variants in echinoderms
Source: R Soc Open Sci. 2015 Dec 4;2(12):150377. doi: 10.1098/rsos.150377 (PMC4807446; doi:10.1098/rsos.150377)
Supplement: Fig.S1.pdf: This is a figure that shows the workflow of the study. [file rsos150377supp1.pdf]

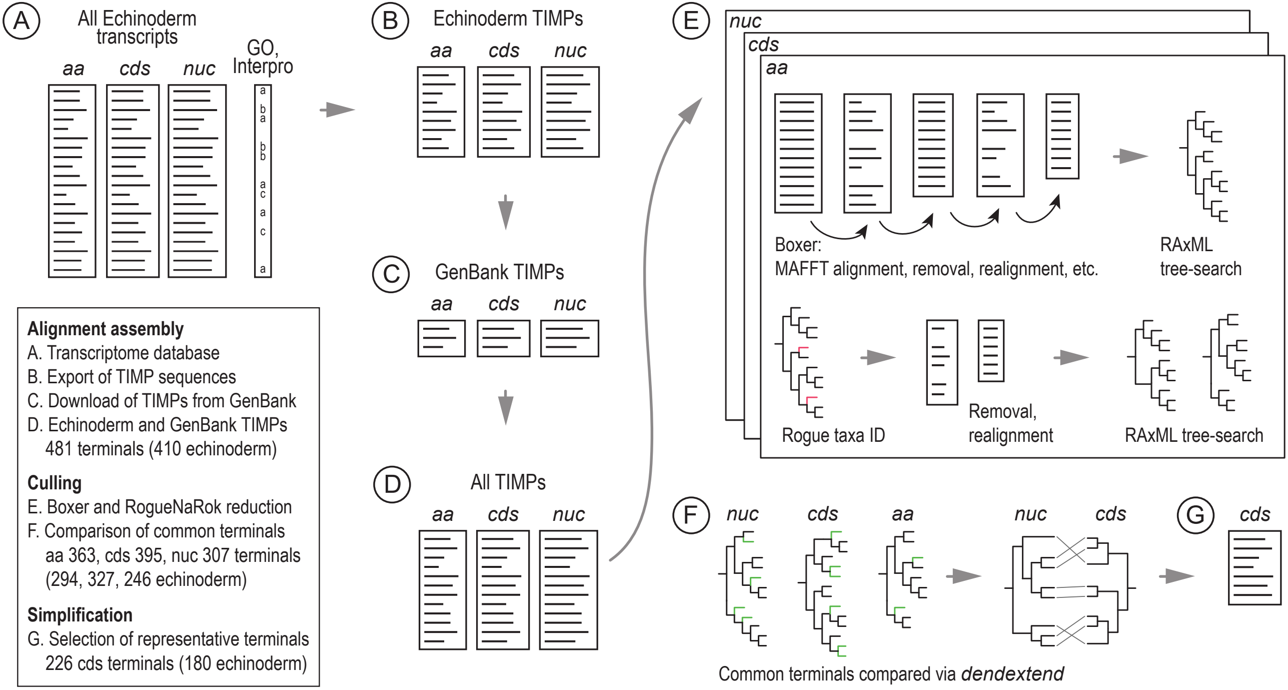

Figure S1. Workflow from echinoderm transcriptome dataset to TIMP phylogeny, showing data sources for figures. An Access database was constructed containing an amino acid sequences ("*aa*"), the underlying coding sequences ("*cds*"), and the original full nuclear sequences ("*nuc*"). These were connected to protein function information from Interpro, PANTHER, and PFam (A), and only those transcripts identified as tissue inhibitors of metalloproteinases (TIMPs) were exported (B). GenBank entries that were identified as TIMPs were exported (C) and combined with echinoderm TIMPs (D). At this stage each of the *aa*, *cds*, and *nuc* had the same terminals. The three sequence sets were then independently run through a custom script, "boxer," which identified possible non-homologous sequences and removed them to improve the alignment. After several rounds of this, a new alignment with fewer terminals was used for tree-searches under maximum likelihood in RAxML. Terminals that were unstable and reduced bootstrap scores were identified with RogueNaRok and removed, the sequences were realigned, and final tree searches under ML in RAxML and parsimony in POY were conducted (E). The different trees from the *aa*, *cds*, and *nuc* alignments were then compared using the "tanglegram" command in the package *dendextend* (F). Terminals were selected to represent all clades and taxa, and some terminals previously culled were added (G); this alignment was translated, aligned, and used to generate the tree shown in Fig. 2.
