# Supplementary material for: Phylotranscriptomic analysis uncovers a wealth of tissue inhibitor of metalloproteinases variants in echinoderms
Source: R Soc Open Sci. 2015 Dec 4;2(12):150377. doi: 10.1098/rsos.150377 (PMC4807446; doi:10.1098/rsos.150377)
Supplement: Fig.S2.pdf: This is a comparison of trees that resulted from searching on alignments of amino acids, coding sequences, and raw nucleotide sequences. [file rsos150377supp2.pdf]

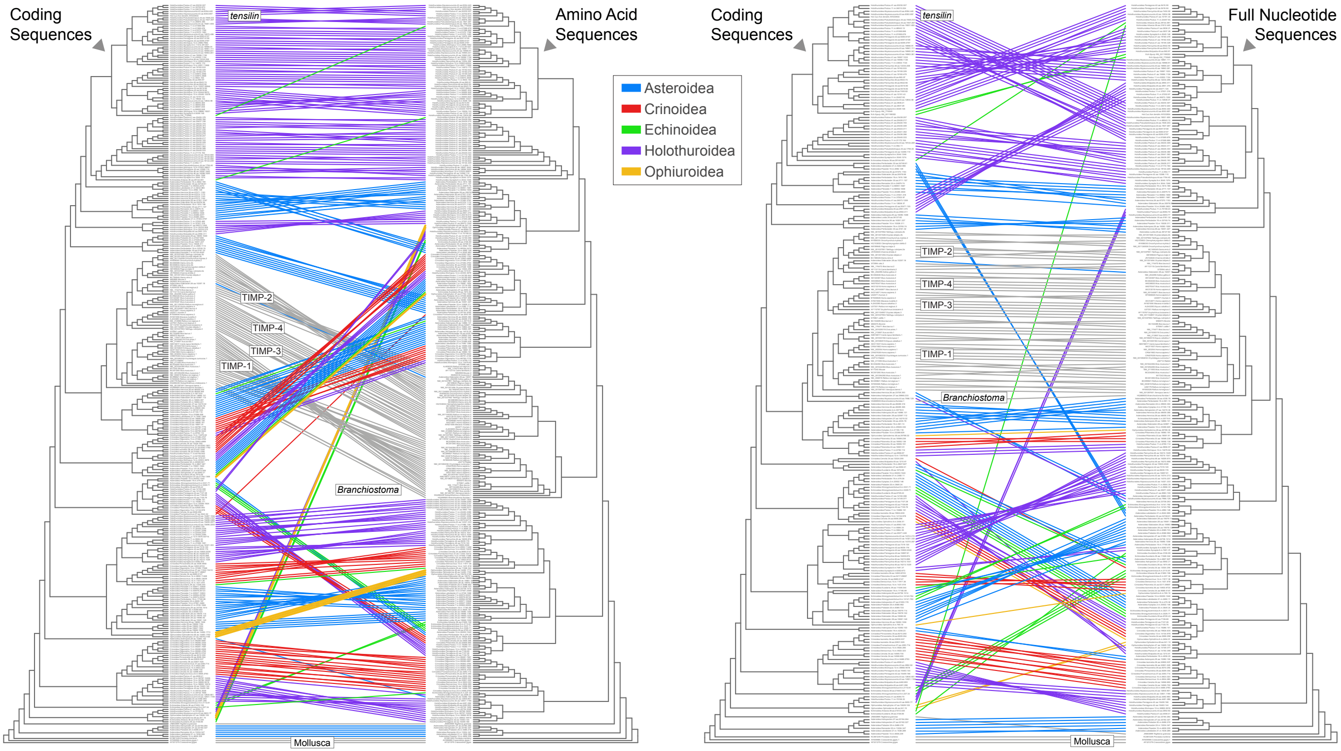

Figure S2. Comparison of common terminals in best trees recovered using different sequence types after alignment and culling. The tree from coding sequences is shown on the left in each comparison, and only terminals shared in both trees are compared.
