# Supplementary material for: Phylotranscriptomic analysis uncovers a wealth of tissue inhibitor of metalloproteinases variants in echinoderms
Source: R Soc Open Sci. 2015 Dec 4;2(12):150377. doi: 10.1098/rsos.150377 (PMC4807446; doi:10.1098/rsos.150377)
Supplement: Fig.S3.pdf: This is the most likely tree found using coding sequences, the alignment of which resulted from our initial alignment-and-culling procedure. [file rsos150377supp3.pdf]

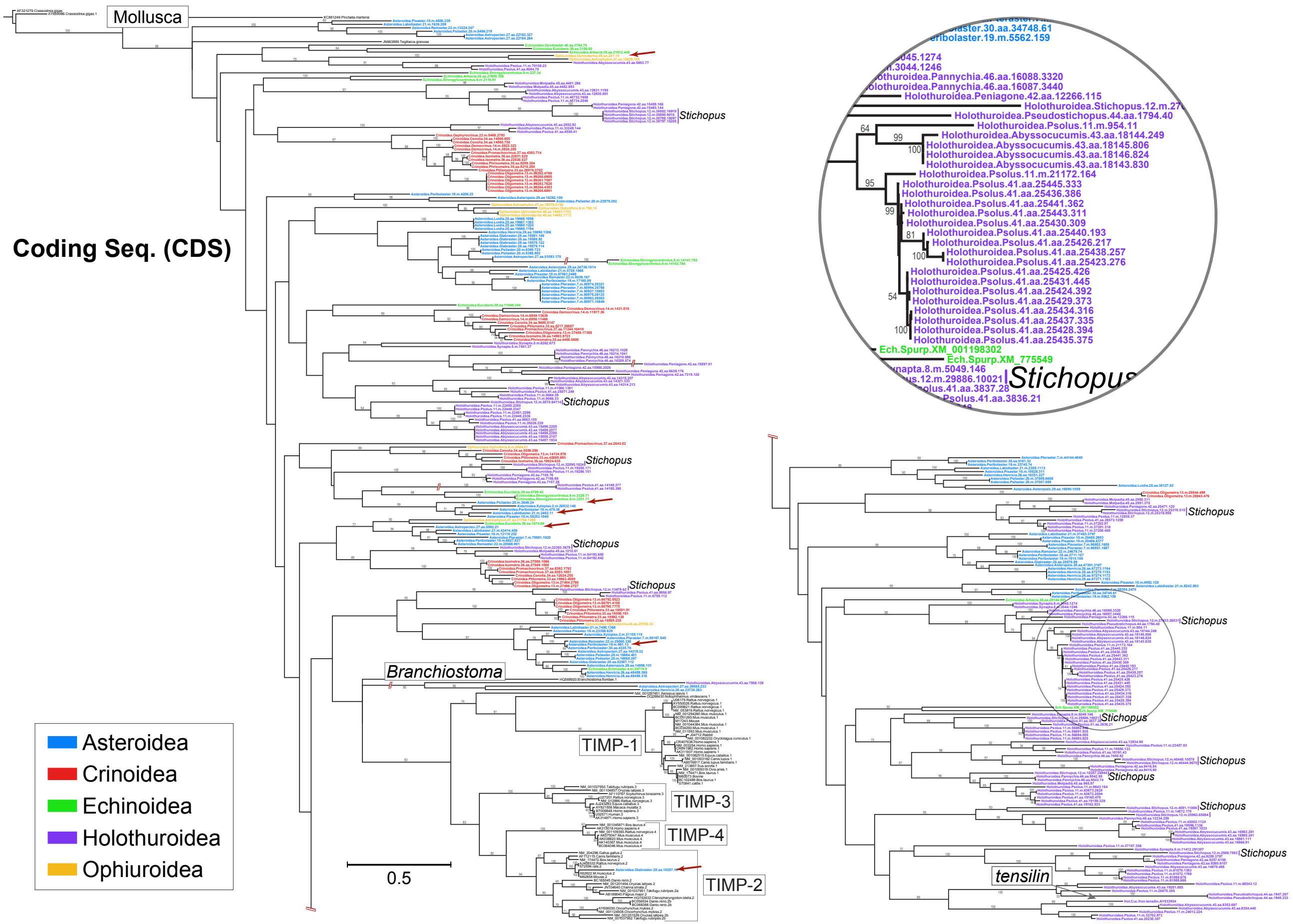

Figure S3. The most likely phylogeny of the TIMP gene family found searching coding sequences under rapid bootstrapping in RAxML. Terminals of interest (molluscan outgroups, the cephalochordate *B. floridae*, vertebrate terminals identified as TIMPs 1–4, and the previously identified echinoderm TIMP tensilin) are noted, and the diversity of TIMPs used by the holothuroid Stichopus is highlighted. Red arrows denote terminals with a read depth of less than 20. Magnified area shows one recent diversification of holothuroid sequences, this in *Psolus*.
